# Double jeopardy: conflict and natural disasters-driven cholera outbreaks in Sudan

**DOI:** 10.1093/inthealth/ihag009

**Published:** 2026-02-27

**Authors:** Rana Osman, Arwa Mohamed, Damilare Akintunde, Alexander Habtemariam, Gorashey Ahmed, Iyas Dawood, Rawa Badri

**Affiliations:** Research and Development, End the Neglect Initiative, Khartoum 1111, Sudan; Faculty of Medicine, Red Sea University, Port Sudan 31111, Sudan; Research and Development, End the Neglect Initiative, Khartoum 1111, Sudan; Sudan Medical Specialization Board, Family Medicine Council, Khartoum 1111, Sudan; Research and Development, End the Neglect Initiative, Khartoum 1111, Sudan; Department of Community Medicine, Faculty of Medicine, Ahmadu Bello University, Zaria 810107, Nigeria; Research and Development, End the Neglect Initiative, Khartoum 1111, Sudan; Faculty of Medicine, University of Global Health, Butaro 6955, Rwanda; Research and Development, End the Neglect Initiative, Khartoum 1111, Sudan; Faculty of Medicine, Omdurman Islamic University, Khartoum 1111, Sudan; Research and Development, End the Neglect Initiative, Khartoum 1111, Sudan; Faculty of Medicine, Omdurman Islamic University, Khartoum 1111, Sudan; Research and Development, End the Neglect Initiative, Khartoum 1111, Sudan; Mycetoma Research Centre, University of Khartoum, Khartoum 1111, Sudan

**Keywords:** cholera, conflict, hygiene, outbreak, sanitation, vaccine

## Abstract

Cholera continues to be a global threat, particularly in regions affected by conflict and environmental shocks, such as Sudan. Such humanitarian crises disrupt sanitation, water, and health systems, exacerbating cholera transmission. Overcrowded camps from displacement, poor hygiene, and weak surveillance further contribute to the outbreaks even beyond national borders. This paper explores armed conflict-driven crises and population movement as epidemiological drivers of cholera. It highlights the critical need for integrated, multisectoral strategies combining vaccination, water, sanitation, and hygiene infrastructure, and early detection systems. The interventions are necessary for mitigating outbreaks and building long-term resilience in such fragile settings.

## Epidemiological drivers of cholera: the role of conflict and population displacement

Cholera is an acute bacterial severe diarrhea, dehydrating disease caused by *Vibrio cholerae* that can be fatal within hours.^1^ The transmission of this disease is facilitated by complex factors, including political conflicts, community movements, and environmental shocks, especially when access to water, sanitation, and hygiene (WASH) facilities is compromised.^2^

The cholera outbreak in Sudan is part of a larger global and regional crisis. Between January 1 and May 25, 2025, the African Region recorded 117 346 cholera cases across 17 countries. The largest burdens were reported in South Sudan (51 054 cases), followed by the Democratic Republic of the Congo (27 008) and Angola (22 557). Sudan reported 16 564 cases during this same period.^2^ From 1 January to 11 August 2025, Sudan reported 48 768 suspected cases of cholera and acute watery diarrhea, with 1 094 deaths, corresponding to a case fatality rate of 2.2%. The disease has affected all 18 states, with notably elevated transmission detected in Khartoum, North Kordofan, White Nile, and across the Darfur region.^3^ Cholera has impacted people of all ages, with more than 70% of reported cases occurring among infants and individuals up to 50 years old.^4^

Armed conflicts and population movements are impacting cholera transmission. Globally, conflicts can lead to the collapse of essential public health infrastructure, including WASH systems.^5^ The subsequent displacement of people into overcrowded camps or urban settings with poor living conditions creates an environment ripe for rapid disease spread.^6^ The ongoing civil war has created one of the world’s most severe humanitarian crises, displacing millions of people internally and across borders, and the movement of displaced people into temporary shelters has created a favorable environment for cholera.^6^

## Health system disruption and response limitations

Approximately 80% of healthcare facilities in conflict-impacted zones are out of service. Medical teams at centers such as Al Jazeera East Hospital in Al Bashraga, Al Jazeera State, continue to care for patients with cholera by delivering oral rehydration therapy and reserving intravenous fluid administration for individuals with severe dehydration.^7^ Before the outbreak of the civil war, Sudan’s healthcare sector was already characterized by inadequate financial investment, a disjointed system structure, and a substantial deficit in health personnel, coupled with pronounced inequalities in the availability, quality, and cost of health services. The nation had been making progress toward achieving the Sustainable Development Goals, although the pace of advancement remained limited. Although Sudan’s Ministry of Health responded promptly to the outbreak, the measures taken have been insufficient in the face of the large surge in cases. In late February, the Ministry of Health, in collaboration with WHO and UNICEF, initiated a vaccination campaign in White Nile State targeting one million individuals. Doctors Without Borders established cholera treatment centers in El Gedaref and later opened another in Atbara, but the need for care has continued to exceed the resources available. Patients and their families have been flocking to nearby health facilities, such as Kosti Teaching Hospital, where shortages of beds have forced staff to manage some cases on the floor. This degree of pressure starkly illustrates the critical need for international assistance and strengthened health-care infrastructure.^8^

## Recommendations for integrated cholera control

1. Oral cholera vaccine implementation should be prioritized in outbreak settings like Sudan, especially in high-risk areas like the Kordofan states, North Darfur, and among the displaced persons population (internally displaced and refugees). Routine immunization should also be addressed where vaccines are available (e.g. measles, Polio vaccine, etc.) to maximize access to the communities.
2. Strengthen access to WASH facilities as a long-term solution to Cholera. This includes the development of piped water systems with water treatment facilities such as chlorination, water filtration, and disinfection; safe water storage at the household level; and the construction of systems for safe sewage disposal, including latrines. Significant priority should be given to displaced population settings. This will serve to reduce cholera transmission.^9^
3. The Sudan government must strengthen integrated disease surveillance (with cholera integrated) and early warning systems in all states (including central Darfur, where no case has been reported there yet), and in displaced persons’ settings, as most states have reported cholera. This will aid in early detection of outbreaks, enhance laboratory capacity through the availability of rapid diagnostic test for early diagnosis, and provide a robust reporting system for suspected cases. Community-based surveillance through volunteers from high-risk communities should be promoted to overcome restrictions caused by conflict.^9,6^
4. Strengthening community awareness and engagement in hotspot and high-risk areas where active conflict and displacement are impacting the spread, and across different groups, including students, religious leaders, volunteers, and vulnerable groups like the displaced populations, through education on cholera prevention.^9,6^ Strengthen structured advocacy to ensure sustained and unhindered humanitarian access across the country, and especially to high-risk areas and to vulnerable groups to secure humanitarian routes and ensure protection of critical infrastructures like water systems and hospital facilities^6^

## Conclusion

In conclusion, this article highlights how conflict, displacement, and a fragile health system have enabled the rapid spread of cholera in Sudan. Despite the prompt response by Sudan’s Ministry of Health, the actions taken have not been sufficient to contain the significant surge in cases.

## Data Availability

No new data new granted or analyzed to support this research.
